# Supplementary material for: Predicting visual function by interpreting a neuronal wiring diagram
Source: Nature. 2024 Oct 2;634(8032):113–23. doi: 10.1038/s41586-024-07953-5 (PMC11446822; doi:10.1038/s41586-024-07953-5)
Supplement: Supplementary file 6 — CRF and ERF predictions for individual TmY4 and TmY9 cells. Analogous to Supplementary Data 3, but for TmY target types. Shown are the top four monosynaptic pathways, the strongest pathway passing through each of the top ten intermediary types (ranking from Extended Data Fig. 7), and the trisynaptic pathway Tm1–TmY–Dm3–TmY (see the section entitled Prediction of spatial normalization). [file 41586_2024_7953_MOESM6_ESM.zip › DataS4/TmY9q/720575940605633894.pdf]

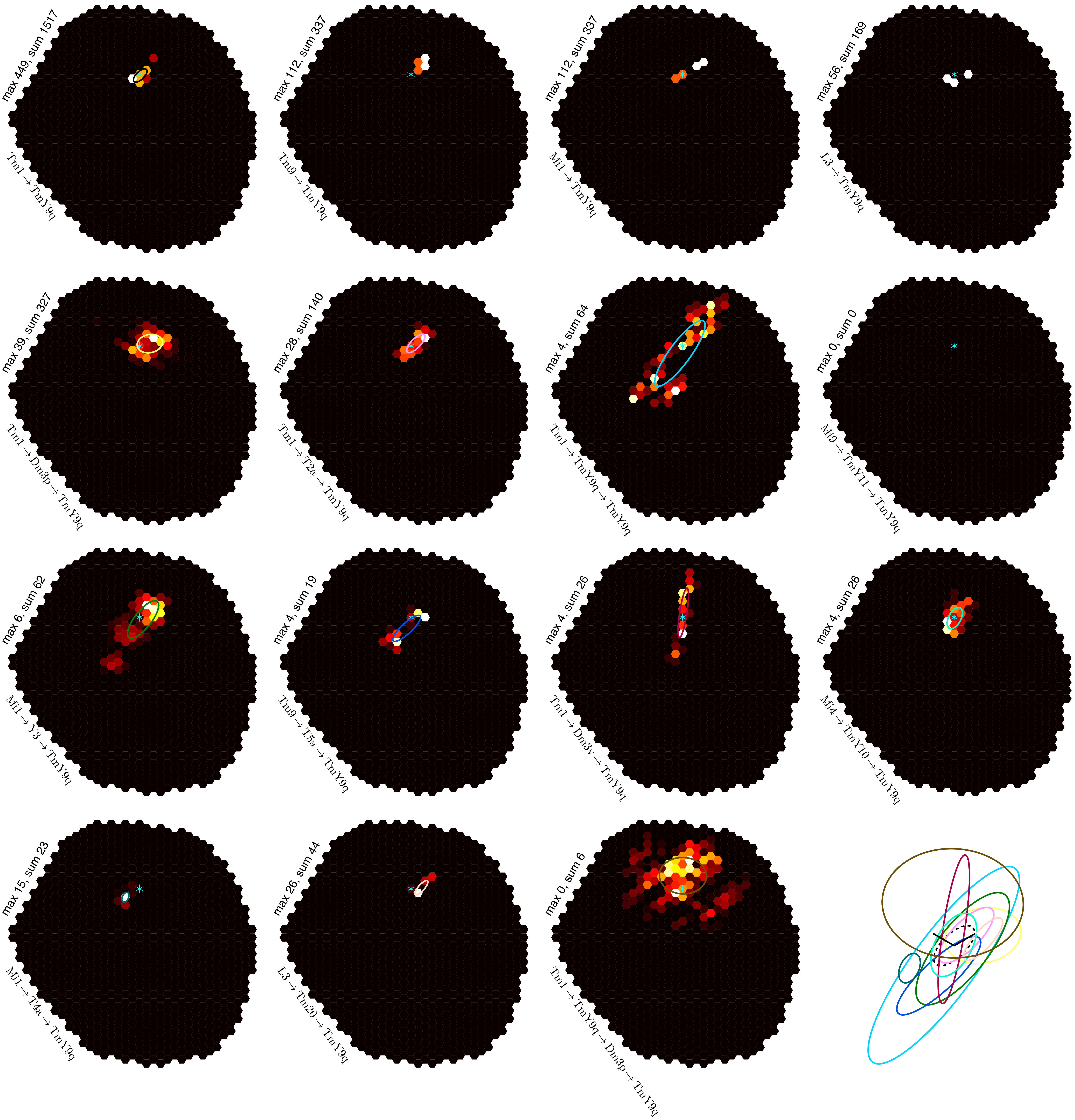

max 449, sum 1517
Tm1 → TmY9q
max 112, sum 337
Tm9 → TmY9q
max 112, sum 337
Mi1 → TmY9q
max 56, sum 169
L3 → TmY9q
max 39, sum 327
Tm1 → Dm3p → TmY9q
max 28, sum 140
Tm1 → T2a → TmY9q
max 4, sum 64
Tm1 → TmY9q → TmY9q
max 0, sum 0
Mi9 → TmY11 → TmY9q
max 6, sum 62
Mi1 → Y3 → TmY9q
max 4, sum 19
Tm9 → T5a → TmY9q
max 4, sum 26
Tm1 → Dm3v → TmY9q
max 4, sum 26
Mi4 → TmY10 → TmY9q
max 15, sum 23
Mi1 → T4a → TmY9q
max 26, sum 44
L3 → Tm20 → TmY9q
max 0, sum 6
Tm1 → TmY9q → Dm3p → TmY9q
